# Prevalence of SARS-CoV-2 Infection in Children and Their Parents in Southwest Germany

**DOI:** 10.1001/jamapediatrics.2021.0001

**Published:** 2021-01-22

**Authors:** Burkhard Tönshoff, Barbara Müller, Roland Elling, Hanna Renk, Peter Meissner, Hartmut Hengel, Sven F. Garbade, Meinhard Kieser, Kathrin Jeltsch, Jürgen Grulich-Henn, Julia Euler, Maximilian Stich, Kristine Chobanyan-Jürgens, Maria Zernickel, Aleš Janda, Lena Wölfle, Thomas Stamminger, Thomas Iftner, Tina Ganzenmueller, Christian Schmitt, Tessa Görne, Vibor Laketa, Sylvia Olberg, Anna Plaszczyca, Mirko Cortese, Ralf Bartenschlager, Constantin Pape, Roman Remme, Daniela Huzly, Marcus Panning, Sebastian Weigang, Sebastian Giese, Kevin Ciminski, Jakob Ankerhold, Georg Kochs, Martin Schwemmle, Rupert Handgretinger, Charlotte M. Niemeyer, Corinna Engel, Winfried V. Kern, Georg Friedrich Hoffmann, Axel R. Franz, Philipp Henneke, Klaus-Michael Debatin, Hans-Georg Kräusslich

**Affiliations:** 1 Department of Pediatrics I, University Children’s Hospital Heidelberg, Heidelberg, Germany; 2 Department of Infectious Diseases, Virology, Heidelberg University, Heidelberg, Germany; 3 Center for Pediatrics and Adolescent Medicine, University Medical Centre and Faculty of Medicine Freiburg, Freiburg im Breisgau, Germany; 4 Institute for Immunodeficiency, University Medical Centre and Faculty of Medicine Freiburg, Freiburg, Germany; 5 University Children’s Hospital Tübingen, Tübingen, Germany; 6 Department of Pediatrics and Adolescent Medicine, Ulm University Medical Center, Ulm, Germany; 7 Institute of Virology, University Medical Centre and Faculty of Medicine, University of Freiburg, Freiburg im Breisgau, Germany; 8 Institute for Medical Biometry and Informatics, Ruprecht-Karls University Heidelberg, Heidelberg, Germany; 9 Department of Clinical Pharmacology and Pharmacoepidemiology, University Hospital Heidelberg, Heidelberg, Germany; 10 Pediatric Clinical-Pharmacological Trial Centre (paedKliPS), University Hospital Heidelberg, Heidelberg, Germany; 11 Institute of Virology, University of Ulm, Ulm, Germany; 12 Institute for Medical Virology, University Hospital of Tübingen, Tübingen, Germany; 13 Department of Infectious Diseases, Molecular Virology, Heidelberg University, Heidelberg, Germany; 14 Heidelberg Collaboratory for Image Processing, Interdisciplinary Centre for Scientific Computing, Heidelberg University, Heidelberg, Germany; 15 European Molecular Biology Laboratory, Heidelberg, Heidelberg, Germany; 16 Centre for Paediatric Clinical Studies at the University Children’s Hospital Tübingen, Tübingen, Germany; 17 Department of Medicine II, Division of Infectious Diseases and Travel Medicine, University Medical Centre Freiburg, Freiburg, Germany

## Abstract

**Importance:**

School and daycare closures were enforced as measures to confine the novel coronavirus disease 2019 (COVID-19) pandemic, based on the assumption that young children may play a key role in severe acute respiratory coronavirus 2 (SARS-CoV-2) spread. Given the grave consequences of contact restrictions for children, a better understanding of their contribution to the COVID-19 pandemic is of great importance.

**Objective:**

To describe the rate of SARS-CoV-2 infections and the seroprevalence of SARS-CoV-2 antibodies in children aged 1 to 10 years, compared with a corresponding parent of each child, in a population-based sample.

**Design, Setting, and Participants:**

This large-scale, multicenter, cross-sectional investigation (the COVID-19 BaWü study) enrolled children aged 1 to 10 years and a corresponding parent between April 22 and May 15, 2020, in southwest Germany.

**Exposures:**

Potential exposure to SARS-CoV-2.

**Main Outcomes and Measures:**

The main outcomes were infection and seroprevalence of SARS-CoV-2. Participants were tested for SARS-CoV-2 RNA from nasopharyngeal swabs by reverse transcription–polymerase chain reaction and SARS-CoV-2 specific IgG antibodies in serum by enzyme-linked immunosorbent assays and immunofluorescence tests. Discordant results were clarified by electrochemiluminescence immunoassays, a second enzyme-linked immunosorbent assay, or an in-house Luminex-based assay.

**Results:**

This study included 4964 participants: 2482 children (median age, 6 [range, 1-10] years; 1265 boys [51.0%]) and 2482 parents (median age, 40 [range, 23-66] years; 615 men [24.8%]). Two participants (0.04%) tested positive for SARS-CoV-2 RNA. The estimated SARS-CoV-2 seroprevalence was low in parents (1.8% [95% CI, 1.2–2.4%]) and 3-fold lower in children (0.6% [95% CI, 0.3-1.0%]). Among 56 families with at least 1 child or parent with seropositivity, the combination of a parent with seropositivity and a corresponding child with seronegativity was 4.3 (95% CI, 1.19-15.52) times higher than the combination of a parent who was seronegative and a corresponding child with seropositivity. We observed virus-neutralizing activity for 66 of 70 IgG-positive serum samples (94.3%).

**Conclusions and Relevance:**

In this cross-sectional study, the spread of SARS-CoV-2 infection during a period of lockdown in southwest Germany was particularly low in children aged 1 to 10 years. Accordingly, it is unlikely that children have boosted the pandemic. This SARS-CoV-2 prevalence study, which appears to be the largest focusing on children, is instructive for how ad hoc mass testing provides the basis for rational political decision-making in a pandemic.

## Key Points

**Question:**

What is the rate of severe acute respiratory syndrome coronavirus 2 (SARS-CoV-2) infections and the seroprevalence of SARS-CoV-2 antibodies in children aged 1 to 10 years and a corresponding parent in a population-based sample in southwest Germany?

**Findings:**

This large-scale, multicenter, cross-sectional investigation of 4964 participants accurately determined anti–SARS-CoV-2 seropositivity by combining the results of enzyme-linked immunosorbent assay and immunofluorescence tests. The estimated SARS-CoV-2 seroprevalence was low in parents (1.8%) and 3-fold lower in children (0.6%).

**Meaning:**

The low seroprevalence of SARS-CoV-2 antibodies in young children in this study may indicate that they do not play a key role in SARS-CoV-2 spreading during the current pandemic.

## Teaser

This cross-sectional investigation conducted in southwest Germany describes the rate of severe acute respiratory syndrome coronavirus 2 infections and the seroprevalence of antibodies in children aged 1 to 10 years, compared with a parent of each child, in a population-based sample.

## Introduction

After emerging in Wuhan, China, in December 2019, severe acute respiratory syndrome coronavirus 2 (SARS-CoV-2) has quickly spread across the globe. A pandemic was declared by the World Health Organization on March 11, 2020. By July 22, 2020, more than 15 million infections had been detected worldwide and the novel coronavirus disease 2019 (COVID-19) had claimed more than 600 000 lives.^1^ Epidemiological data from the early stage of the pandemic suggested that children show a much milder disease course than adults.^2,3,4,5,6^ In addition, it matches the epidemiological features of the 2002-2003 SARS pandemic.^7^ Overall, children younger than 18 years are thought to account for only 1% to 2% of detected COVID-19 cases worldwide.^8^

Importantly, low detection rates of SARS-CoV-2 RNA in children do not necessarily rule out that children are relevant drivers of transmission. There are 2 possible scenarios: (1) infants and children may display a reduced susceptibility to infection compared with adults and (2) the mild or asymptomatic disease manifestation in children escapes detection, resulting in an underestimation of the real rate of infection. The mild clinical presentation or asymptomatic nature of pediatric SARS-CoV-2 infections could be a hidden driver of the pandemic, especially when limited test capacities introduce a bias toward more severe cases.^9^

An earlier report from China investigating a cohort with 391 COVID-19 cases and 1286 close contacts suggested that children were as likely to be infected as adults.^10^ On the other hand, a large study from Iceland found a lower incidence of acute SARS-CoV-2 infection in children than adults.^11^ Likewise, a serological study from Switzerland observed a lower SARS-CoV-2 seroprevalence in young children (5-9 years) compared with adults.^12^ A lower risk of infection for children was also reported in a study investigating transmission dynamics within families in Israel, a survey on SARS-CoV-2 infection by nasopharyngeal swabs in the municipality of Vo', Italy, and in a population-based seroepidemiological study in Spain.^13,14,15^ However, most of the aforementioned studies suffered from a relatively small sample size, particularly with respect to young children. Accordingly, a solid basis for the implementation of far-reaching measures, such as closures of elementary schools and childcare facilities, for transmission control of the COVID-19 pandemic seems to still be missing. The pandemic has severe and multifaceted consequences for young children, including mental health threats, delayed or halted progress in school education, complications through delays of necessary medical care, malnutrition, poverty, and domestic violence. Thus, a better understanding of their role in the pandemic is of vital importance to justify their separation from educational and social activities in kindergartens and schools.

To address this important issue, we performed a large-scale, multicenter, cross-sectional study on the point prevalence of SARS-CoV-2 infections and seroprevalence in a paired parent-child study design in the federal state of Baden-Württemberg in southwest Germany, the region with the second-highest case numbers of COVID-19 in Germany (337 cases per 100 000 inhabitants) at initiation of this study. The following study objectives were investigated. First, what is the rate of SARS-CoV-2 infections and the seroprevalence of SARS-CoV-2 antibodies in children aged 1 to 10 years and 1 corresponding parent of each child in a population-based sample in southwest Germany? Second, are there age-dependent subgroups concerning seroprevalence in children? Third, are family size, the attendance of exceptional child daycare facilities (emergency child daycare during government-imposed COVID-19 lockdown), or previous contact with a person with COVID-19 associated with the risk of infection?

## Methods

### Study Design and Conduct

This was a noninterventional, uncontrolled, open, national, multicenter, cross-sectional study on the point prevalence of SARS-CoV-2 infections as determined by reverse transcription–polymerase chain reaction (RT-PCR) testing of nasopharyngeal and oropharyngeal swabs and presence of SARS-CoV-2 antibodies in serum (German Registry for Clinical Studies Identifier 00021521). The 4 study centers were the University Children’s Hospitals in Freiburg, Heidelberg, Tübingen, and Ulm, Germany. Participants were recruited through public announcements of the parent-child study in national and local newspapers as well as social media networks between April 22 and 30, 2020. Study participation on application was random and voluntary. Participants were investigated during the period from April 22 to May 15, 2020. The study was designed, analyzed, and reported according to the Strengthening the Reporting of Observational Studies in Epidemiology (STROBE) reporting guideline (https://www.strobe-statement.org).

The study protocol was approved by the independent ethics committees of each center. The study was conducted according to the Declaration of Helsinki. Written informed consent was obtained from all parents/guardians, with assent from children when appropriate for their age.

### Eligibility Criteria and Study Procedure

Participants were eligible for enrollment if they met the following inclusion criteria: (1) 1 child (male or female) aged 1 to 10 years, (2) 1 corresponding parent (male or female) without an age limit (the parents decided among themselves who would participate), (3) residency of the child and parent in the same household, (4) residency in the state of Baden-Württemberg, and (5) written consent to the study. Key exclusion criteria were (1) severe congenital disease (eg, infantile cerebral palsy, severe congenital malformation), (2) congenital or acquired immunodeficiency, and (3) laboratory-confirmed SARS-CoV-2 infection in the child or participating parent before study enrollment.

Study participants received a questionnaire on occupation, age, and chronic illnesses of the participating parent. Details are given in the eMethods in the Supplement.

### Laboratory Analysis

Specimens for polymerase chain reaction (PCR) diagnostics were collected as oropharyngeal and nasopharyngeal swabs in eSwab (Copan) or Sigma-Virocult (MWE), respectively. Experimental details of RT-PCR reactions are in the eMethods in the Supplement.

The applied CE–in vitro diagnostic certified serological tests for SARS-CoV-2 have a high specificity according to the manufacturer’s documentation (Euroimmun SARS-CoV-2 IgG enzyme-linked immunosorbent assay [ELISA]: 99.6%, validated on 1344 samples; Roche Elecsys Anti-SARS-CoV-2 test: 99.81%, validated on 10 533 samples; Mikrogen recomWell ELISA: 98.7%, validated on 300 samples). With specificities less than 100%, however, false-positive results constitute a substantial proportion of all positive results in populations with a low seroprevalence. To increase specificity, both an ELISA test against the viral S1 protein as well as an immunofluorescence test on SARS-CoV-2–infected VeroE6 cells were performed for all samples. Discordant results were clarified by electrochemiluminescence immunoassays, a second ELISA, or an in-house Luminex-based assay.^17^ We had previously determined that a combination of these complementary assays considerably increases the specificity of the results without compromising the sensitivity.^16^ The workflow of the serological analyses is shown eTable 1 in the Supplement; details of the serological analyses, the immunofluorescence assay, and the neutralization assay are given in the eMethods in the Supplement.

### Statistical Analysis

Analyses were performed using R version 4.0.0 (R Foundation for Statistical Computing). Results for continuous variables are presented as medians with interquartile ranges (IQR) and minimum and maximum values, unless stated otherwise. Generalized linear mixed-effects models with a logit link function were used to evaluate the association between the odds of SARS-CoV-2 seropositivity and the covariates of age, number of siblings in the family, sex, exceptional child daycare, and previous contact with a person with COVID-19. To cope with the paired structure between child and parent within 1 family, models with the random factor of family were computed. Three mixed-effects logistic regression models with the response seropositivity were computed. First, a mixed-effects logistic regression with the covariate parent, a reference (intercept) of child, and a random intercept of family was calculated. Model 2 included the covariate of age group (children aged 1 to 5 years, children aged 6 to 10 years, and parents), and model 3 included the covariates of age group (3 levels), number of siblings in the family, sex, attendance at exceptional child daycare, and previous, noticed contact with a person with COVID-19 (eTable 2 in the Supplement). Function glmer from the package lme4, version 1.1-23, was used to fit the mixed-effects logistic regression models. Estimated seroprevalence for age groups was derived from fitted mixed-effects logistic regression, and 95% CIs for prevalence were computed using a bootstrap approach based on 600 replications, each with a sample size of 1500 sampled from entire study population (package boot, version 1.3-25). The mid-*P* McNemar test was used to compare the ratio between a parent who was seronegative with a child with seropositivity and a parent with seropositivity with a child who was seronegative (R package Exact, version 2.0). We compared potential COVID-19–associated symptoms (eg, fever, cough, diarrhea, dysgeusia) between individuals who were seronegative vs seropositive with the Boschloo test (R package Exact, version 2.0). No a priori formulated hypotheses were tested, and therefore all *P* values and CIs are reported as descriptive measures.

## Results

### Descriptive Characteristics of Study Population

Of the 2550 child-parent pairs enrolled, 2482 pairs were available for final analysis (eFigure 1 in the Supplement). A total of 2482 children (median age, 6 [range, 1-10] years; 1265 boys [51.0%]) and 2482 parents (median age, 40 [range, 23-66] years; 615 men [24.8%]) were included. Demographic statistics on these 4964 study participants are given in Table 1. Further data are given in the eResults and eFigure 2 in the Supplement.

**Table 1.** Demographics of the Study Population^a^

Characteristic	Participants, No. (%)			
	Parents	Children	Subgroup age range, y	
			1-5	6-10
Total No.	2482	2482	1129 (45.5)	1353 (54.5)
Age, median (range) [IQR], y	40 (23-66) [36-43]	6 (1-10) [4-8]	3 (1-5) [2-4]	8 (6-10) [7-9]
Sex				
Male	615 (24.8)	1265 (51.0)	567 (22.8)	698 (28.1)
Female	1857 (74.8)	1175 (47.3)	542 (21.8)	633 (25.5)
Not documented	10 (0.4)	42 (1.7)	20 (0.8)	22 (0.9)
Region				
Freiburg	532 (21.4)	532 (21.4)	255 (10.3)	277 (11.2)
Heidelberg	669 (27.0)	669 (27.0)	289 (11.6)	380 (15.3)
Tübingen	588 (23.7)	588 (23.7)	291 (11.7)	297 (12.0)
Ulm	693 (27.9)	693 (27.9)	294 (11.8)	399 (16.1)
Previous contact with person with coronavirus disease 2019	237 (10.7)	102 (4.3)	56 (2.4)	43 (1.8)
Unknown, No.	22	24	NA	NA
Attending exceptional child daycare^b^	NA	583 (31.0)	298 (12.1)	285 (11.6)
Unknown, No.	NA	17	10	7

Abbreviations: IQR, interquartile range; NA, not applicable.

^a^ Data are given as median (range) and interquartile range, if not stated otherwise.

^b^ The term *exceptional child daycare* refers to emergency child daycare during government-imposed lockdown because of the coronavirus disease 2019 pandemic.

### Detection of Acute SARS-CoV-2 Infection by RT-PCR

Among the 4964 persons tested by RT-PCR, only 2 participants (0.04%), 1 child and the corresponding parent, tested positive for SARS-CoV-2 RNA. Both participants reported having had mild symptoms consistent with COVID-19 several weeks before but were asymptomatic at study entry, which was consistent with the presence of neutralizing antibodies in both participants.

### Rate of SARS-CoV-2 Seropositivity Among Samples

Table 2 summarizes the results of the serological analyses. All serum samples that were scored negative in both initial assays (n = 4785) were classified as IgG negative and not further analyzed. A total of 60 samples were found to be positive in both assays and classified as positive; in all cases, this assessment could be confirmed by a third assay. A number of samples (n = 119) displayed borderline reactivity in ELISA testing or yielded discordant results between ELISA and immunofluorescence tests.^16^ In these cases, at least 1 of the additional tests was used for final classification. As shown in Table 2, only a small proportion of the samples that had positive results in only 1 of the initial assays was finally confirmed as positive (1 [3.8%] via ELISA only and 4 [8.5%] via immunofluorescence only); the confirmed proportion was also small for samples that had borderline results in ELISA tests and negative results in immunofluorescence tests (2 [4.8%]). Hence, in the remaining discordant samples (>90%), the positive readout had represented a false-positive detection in the respective assay.

**Table 2.** Positive Reactivity of Specimens in the Serological Assays

Initial result		Identified, No.	Confirmed, No. (%)
ELISA	Immunofluorescence		
Positive	Positive	60	60 (100.0)
Borderline	Positive	4	3 (75.0)
Positive	Negative	26	1 (3.8)
Borderline	Negative	42	2 (4.8)
Negative	Positive	47	4 (8.5)
Total		179	70 (39.1)

Abbreviation: ELISA, enzyme-linked immunosorbent assay.

### Neutralizing Antibody Positivity

All 70 serum samples that had been classified as clearly IgG positive were further characterized for the presence of neutralizing antibodies in titration experiments, using infection of VeroE6 cells with SARS-CoV-2 (BavPat strain) as the readout. Virus-neutralizing activity could be confirmed for 66 of 70 IgG-positive serum samples (94.3%).

### SARS-CoV-2 Seroprevalence Per Age Group

Of 4964 persons tested for SARS-CoV-2 IgG antibodies, 70 participants were categorized as seropositive (1.4%). Altogether, 22 of 2482 children (0.9%) and 48 of 2482 parents (1.9%) were seropositive. The number of individuals who were seropositive in the age group 1 to 5 years was 9 of 1129 (0.8%); in the age group of 6 to 10 years, it was 13 of 1353 (1.0%), and in the parent group, it was 48 of 2482 (1.9%).

Reported potential COVID-19–associated symptoms that had occurred since the end of February 2020 are shown in Table 3, stratified according to SARS-CoV-2 antibody status. Unlike children, parents with earlier symptoms compatible with COVID-19 were more often antibody positive. The SARS-CoV-2 seroprevalence stratified according to the characteristic of exceptional child daycare is shown in eTable 3 in the Supplement. In 580 of 2482 children (23.4%) who attended exceptional child daycare, the seropositivity rate in children (3 of 580 [0.5%]) was not significantly different from those not attending (19 of 1863 [1.0%]).

**Table 3.** Potential Coronavirus Disease 2019–Associated Symptoms Reported by Parents or Children

Patient symptoms	Participants, No./total No. (%)			*P* value
	Overall	Seropositive	Seronegative	
**Parents**				
Fever				
Yes	347/2482 (14.0)	21/48 (43.8)	326/2434 (13.4)	<.001
No	2135/2482 (86.0)	27/48 (56.2)	2108/2434 (86.6)	
Cough				
Yes	863/2482 (34.8)	28/48 (58.3)	835/2434 (34.3)	<.001
No	1619/2482 (65.2)	20/48 (41.7)	1599/2434 (65.7)	
Diarrhea				
Yes	387/2482 (15.6)	11/48 (22.9)	376/2434 (15.4)	.15
No	2095/2482 (84.4)	37/48 (77.1)	2058/2434 (84.6)	
Dysgeusia				
Yes	148/2482 (6.0)	23/48 (47.9)	125/2434 (5.1)	<.001
No	2334/2482 (94.0)	25/48 (52.1)	2309/2434 (94.9)	
**Children**				
Fever				
Yes	648/2482 (26.1)	8/22 (36.4)	640/2460 (26.0)	.32
No	1834/2482 (73.9)	14/22 (63.6)	1820/2460 (74.0)	
Cough				
Yes	870/2482 (35.1)	4/22 (18.2)	866/2460 (35.2)	.11
No	1612/2482 (64.9)	18/22 (81.8)	1594/2460 (64.8)	
Diarrhea				
Yes	321/2482 (12.9)	5/22 (22.7)	316/2460 (12.8)	.19
No	2161/2482 (87.1)	17/22 (77.3)	2144/2460 (87.2)	
Dysgeusia				
Yes	21/2482 (0.9)	0	21/2460 (0.9)	>.99
No	2461/2482 (99.1)	22/22 (100)	2439/2460 (99.1)	

### Parent-Child Concordance in Seropositivity

Table 4 shows the seroprevalence of children and parents within the same family. Altogether, there were 56 families with at least 1 child or parent with seropositivity. There were 14 child-parent pairs in which both members were seropositive, while 34 parents who were seropositive had a child who was seronegative, and 8 children who were seropositive had a parent who was seronegative (Table 4). The combination of a parent who was seropositive and a corresponding child who was seronegative (n = 34) was 4.3 (95% CI, 1.19-15.52) times more common than the combination of a parent who was seronegative and a corresponding child who was seropositive (n = 8) (*P* < .001).

**Table 4.** Number of Children and Their Corresponding Parent With Seropositivity and Seronegativity

Test result	All children		Children age range, y			
			1-5		6-10	
	Parent with negative result	Parent with positive result	Parent with negative result	Parent with positive result	Parent with negative result	Parent with positive result
**Child**						
Negative	2426	34	1105	15	1321	19
Positive	8	14	4	5	4	9
Mid-*P* McNemar test	<.001		.02		.003	

Three mixed-effects logistic regression models with the response seropositivity were computed. Model 1 yielded an estimated prevalence for children aged 1 to 10 years of 0.6% (95% CI, 0.3-1.0; Table 5). This estimated seroprevalence was 3-fold lower than in parents (1.8% [95% CI, 1.2-2.4]; Table 5). eTable 4 in the Supplement shows covariates of models 2 and 3 stratified according to seropositivity. In model 2 (log odds, 5.51 [95% CI, 3.01-7.87]) and model 3 (log odds, 5.15 [95% CI, 2.59-7.68]), only the variable age group was significantly associated with SARS-CoV-2 seropositivity, indicating that the rate of children aged 1 to 5 years who were seropositive was lower than the rate of parents who were seropositive (eTable 2 in the Supplement).

**Table 5.** Number of Participants Who Were Seropositive, Estimated Mean Family Seroprevalence, and 95% CIs^a^

Group	Total participants, No.	Participants with seropositivity, No.	Estimated prevalence of seropositivity, % (95% CI)
**Children age range, y**			
1-5	1129	9	0.5 (0.2-0.9)
6-10	1353	13	0.7 (0.4-1.4)
Children	2482	22	0.6 (0.3-1.0)
Parents	2482	48	1.8 (1.2-2.4)

^a^ The results for the children group were estimated from mixed-effect logistic regression model 1; the results for the groups children aged 1 to 5 years, children aged 6 to 10 years, and parents were estimated from mixed-effect logistic regression model 2.

## Discussion

The key results of this large SARS-CoV-2 prevalence study in children and a corresponding parent are the following: only 0.04% of study participants tested positive for SARS-CoV-2 RNA. The estimated SARS-CoV-2 seroprevalence was low in parents (1.8% [95% CI, 1.2-2.4%]) and even lower (3-fold lower) in children aged 1 to 10 years (0.6% [95% CI, 0.3-1.0%]). Hence, the prevalence of previously undiagnosed SARS-CoV-2 infections before and during the lockdown in southwest Germany was very low. The lower SARS-CoV-2 seroprevalence in young children compared with their corresponding parent is an important observation, because it indicates that children are very unlikely to have boosted the COVID-19 outbreak in southwest Germany during the period of investigation. This contrasts with other respiratory tract infections, such as influenza or pneumococci, in which children can play a prominent role for the dissemination of the disease.^18,19^ The higher frequency and proximity of social contacts of children are considered to be a major driver of virus transmission.^20,21^ Given these theoretical concerns, the provision of real-life data on the low SARS-CoV-2 attack rate in young children during a running pandemic is important. These data contributed to the political decision to allow for a stepwise reopening of childcare facilities and schools after the general lockdown in southwest Germany.

To yield robust data on circulating SARS-CoV-2 antibodies, this study used an elaborate combination of serological tests (ELISAs and immunofluorescence testing). The accuracy of serological SARS-CoV-2 studies crucially depends on the sensitivity and specificity of the assay or assays used.^22,23^ Whereas many of the initial studies have used assays with low specificity and/or sensitivity, test systems with better validation and specification became available in the past months.^24,25^ The CE–in vitro diagnostic–certified ELISA test used as an initial test in this study has previously been used in serological surveys.^12^ The manufacturer has reported a specificity of 99.6% (based on 1344 control samples) and a sensitivity of 94.4% for serum samples from patients with COVID-19 at day 10 or later after symptom onset (n = 72). Studies by our team and others found a slightly lower specificity when including borderline results (eg, 96.3% or 98.6%).^16,26^ Given the low SARS-CoV-2 prevalence in the general population, when this study was performed, test combinations with specificities greater than 99% are required to obtain valid results. A combination of 2 different tests improves accuracy markedly and has recently been used in a large seroepidemiological study.^15^ Here, we combined ELISA with immunofluorescence as complementary tests for all samples.^16^ Moreover, we further analyzed discordant samples by additional tests to maximize both specificity and sensitivity. As shown in Table 2, both initial assays independently yielded 40% to 50% of false-positive results, but these could be eliminated by a combination of the 2 readouts. Likewise, each assay by itself yielded false-negative results, which could be largely corrected for by the combination of 2 readouts and further assessment of discordant findings by 1 or more additional assays. We are therefore confident that the serological data presented are highly accurate. Four of the 70 serum samples clearly classified as seropositive did not display neutralizing activity in titration experiments in tissue culture. However, we also observed lack of neutralizing antibodies in patients who were seropositive with confirmed SARS-CoV-2 infection. This observation underscores that at the current state of knowledge, antibody levels detected by serological assays must not be interpreted as correlates for the presence of neutralizing antibodies or protective immunity.^25^

The observation that attendance of exceptional child daycare facilities was not associated with SARS-CoV-2 seropositivity is important, because it indicates that attendance of exceptional child daycare facilities does not necessarily boost SARS-CoV-2 spreading, despite the fact that hygiene practices in these young children are difficult to achieve. The age of the study participants was associated with SARS-CoV-2 seropositivity; the estimated prevalence of seropositivity in children was 3-fold lower than in their corresponding parents. The observation of a lower susceptibility toward SARS-CoV-2 infections in young children is consistent with previous studies.^12,14,15^ This variable susceptibility to SARS-CoV-2 is still not understood. Potential explanations include the lower expression levels of angiotensin-converting enzyme 2, the cellular entry receptor of SARS-CoV-2 in the nasal epithelium of children.^27^ Natural resistance to SARS-CoV-2 may originate from the abundance of IgM memory B cells in children and cross-priming by distant respiratory viruses or environmental antigens.^28^ More specifically, seasonal coronaviruses may provide some protection, as indicated by SARS-CoV-2-reactive CD4+ T cells in up to 60% of individuals without exposure.^29,30^

### Strengths

The strengths of this study are its multicenter and paired child-parent design and the high number of study participants. In addition, it features the combination of serological assays for achieving highest accuracy in the determination of circulating SARS-CoV-2 antibodies, which is paradigmatic for serological studies in areas with low SARS-CoV-2 prevalence.

### Limitations

A potential limitation is the voluntary study participation. However, the inclusion of 4 geographically separated towns and their adjacent regions and the high number of study participants at least partially compensates for this shortcoming and allows for drawing general conclusions on the role of children in the pandemic. A limitation of the statistical analysis is that because of missing values, the results of logistic regression model 3 are based on a lower number of observations and families than models 1 and 2. Moreover, because the study was performed during lockdown, we cannot exclude that the lower rate of children who were seropositive compared with adults partly results from reduced exposure. Since we did not observe a higher rate of seropositivity among children attending exceptional child daycare facilities, we are confident that the contact restrictions of children do not explain this striking difference of seropositivity between children and adults. Because only young children were included, no inference can be made about older children. Finally, while the data suggest that children are less likely to get infected, the study design does not allow drawing conclusions on the infectivity of a child infected with SARS-CoV-2.

## Conclusions

In this cross-sectional study, the spread of undiagnosed SARS-CoV-2 infections during the initial phase of SARS-CoV-2 dissemination and the subsequent lockdown period in southwest Germany was low. Children aged 1 to 10 years appeared not to be particular drivers of the pandemic. Overall, this large SARS-CoV-2 prevalence study in children is instructive for how ad hoc mass testing provides the basis for rational political decision-making in a pandemic setting.

## Notes

Supplement. **eMethods.** Questionnaire for study participants, RNA isolation from nasal and oropharyngeal swabs and reverse-transcriptase polymerase chain reaction (RT-PCR), serological analysis, and determination of neutralizing antibody titers (NT) **eResults.** Descriptive characteristics of study population **eTable 1.** Workflow of the serological analyses. **eTable 2.** Summary of mixed effect logistic regression models with the dependent variable “seropositivity” **eTable 3.** SARS-CoV-2 seroprevalence stratified according to the characteristic “exceptional child day care” **eTable 4.** Covariates stratified according to seropositivity **eFigure 1.** Disposition of study participants **eFigure 2.** Region of residence of study participants analyzed according to the three-digit postal code
