# Supplementary material for: Prevalence of SARS-CoV-2 Infection in Children and Their Parents in Southwest Germany
Source: JAMA Pediatr. 2021 Jan 22;175(6):1–8. doi: 10.1001/jamapediatrics.2021.0001 (PMC7823424; doi:10.1001/jamapediatrics.2021.0001)
Supplement: Supplement. — eMethods. Questionnaire for study participants, RNA isolation from nasal and oropharyngeal swabs and reverse-transcriptase polymerase chain reaction (RT-PCR), serological analysis, and determination of neutralizing antibody titers (NT) eResults. Descriptive characteristics of study population eTable 1. Workflow of the serological analyses. eTable 2. Summary of mixed effect logistic regression models with the dependent variable “seropositivity” eTable 3. SARS-CoV-2 seroprevalence stratified according to the characteristic “exceptional child day care” eTable 4. Covariates stratified according to seropositivity eFigure 1. Disposition of study participants eFigure 2. Region of residence of study participants analyzed according to the three-digit postal code [file jamapediatr-e210001-s001.pdf]

Supplemental Online Content

Tönshoff B, Müller B, Elling R, et al. Prevalence of SARS-CoV-2 infection in children and their parents in southwest Germany. *JAMA Pediatr.* Published online January 22, 2021. doi:10.1001/jamapediatrics.2021.0001

eMethods. Questionnaire for study participants, RNA isolation from nasal and oropharyngeal swabs and reverse-transcriptase polymerase chain reaction (RT-PCR), Serological analysis, and Determination of neutralizing antibody titers (NT)

eResults. Descriptive characteristics of study population

eTable 1. Workflow of the serological analyses.

eTable 2. Summary of mixed effect logistic regression models with the dependent variable "seropositivity"

eTable 3. SARS-CoV-2 seroprevalence stratified according to the characteristic "exceptional child day care"

eTable 4. Covariates stratified according to seropositivity

eFigure 1. Disposition of study participants

eFigure 2. Region of residence of study participants analyzed according to the three-digit postal code

This supplemental material has been provided by the authors to give readers additional information about their work.

© 2021 American Medical Association. All rights reserved.

eMethods. Questionnaire for study participants, RNA isolation from nasal and oropharyngeal swabs and reverse-transcriptase polymerase chain reaction (RT-PCR), Serological analysis, and Determination of neutralizing antibody titers (NT)

Questionnaire for study participants
Questions concerning the children included (i) chronic illnesses, (ii) attendance of day-care centers, kindergarten or elementary school, (iii) the length of time the children had been in home care or whether they continued to attend day-care centers, kindergarten or after-school care within the framework of exceptional child day care. For both children and parents, we inquired the following items (i) Contact to someone with laboratory-confirmed SARS-CoV-2 infection, (ii) prior diagnosis of SARS-CoV-2 infection in the participants themselves (exclusion criterion), or (iii) whether potential COVID19-associated symptoms (fever, cough, "common cold", diarrhea or loss of smell and taste) had occurred since the end of February 2020 (outbreak of the COVID-19 pandemic in Europe).

Specimens for PCR diagnostics
Specimens for PCR diagnostics were collected as oropharyngeal and nasopharyngeal swabs in eSwab, Copan, Milan, Italy or Sigma-Virocult, respectively. Collected samples were transported in sterile containers, delivered to the diagnostic laboratory within a few hours, and examined directly or stored at 4°C until further processing. Blood samples for serological analyses were centrifuged and serum was collected. Serum samples were either analyzed directly or stored at 4°C until further processing. Serum shipment to test centers at other sites was by overnight courier at room temperature.

RNA isolation from nasal and oropharyngeal swabs and reverse-transcriptase polymerase chain reaction (RT-PCR)
For samples from Heidelberg, RNA was isolated from nasopharyngeal swabs using QIAGEN Kits (QIAGEN, Hilden, Germany) automated on the QIASymphony instrument (DSP Virus pathogen mini kits), and eluted in 115 µL elution buffer. Ten µl of extracted samples were used in a 20 µL RT-PCR reaction, carried out using various reagent mixes: LightMix Modular SARS and Wuhan CoV E-gene, LightMix Modular SARS and Wuhan CoV N-gene, LightMix Modular Wuhan CoV RdRP-gene and LightMix Modular EAV RNA Extraction Control (TIBMOLBIOL, Berlin, Germany) and LightCycler Multiplex RNA Virus Master (Roche, Mannheim, Germany) according to manufacturer's instructions. RT-PCR was performed on LightCycler 480 instruments (Roche, Mannheim, Germany). Thermal profile was as follows: reverse transcription step at 55 °C for 5 min, followed by denaturation at 95°C for 5 min, and 45 amplification cycles (denaturation at 95°C for 5 sec, annealing at 60°C for 15 sec, and elongation at 72°C for 15 sec). For samples from Freiburg, reactions were performed on an Abbott *m*2000 platform with automated extraction of nucleic acids using the total nucleic acid sample preparation kit in batches of up to 96 samples. The Real Time SARS-CoV-2 assay utilizes two real-time detection probes: one probe combined for the N and RNA dependent RNA polymerase genes, and a second probe for the internal control to assess overall performance, including nucleic acid extraction and possible PCR inhibition. Automated extraction was performed using a sample input volume of 500 µL viral transport medium, followed by automated addition of amplification pack reagents and extracts (40 µL volume used for PCR amplification and detection). Positive and negative controls provided by the manufacturer were included with each run of patient samples. Amplification curves were interpreted by the *m*2000rt system. Results were reported as detected or not detected.

Serological analysis
ELISA determination of reactivity against the S1 domain of the viral spike protein was carried out using the Euroimmun Anti-SARS-CoV-2-ELISA (IgG) test kit (Euroimmun AG, Lübeck, Germany, EI 2606-9601 G) according to the manufacturer’s protocol. Samples were processed on a Euroimmun Analyzer I instrument and interpretation of results was performed according to the test manufacturer’s instructions (ratio values 1.1 and higher = positive; 0.8 − 1.1 = borderline). Measurements for determination of reactivity against the viral nucleocapsid (N) protein were carried out using either the Elecsys® Anti-SARS-CoV-2 test kit (#09 203 095 190, Roche Diagnostics, Mannheim) processed on a Roche Cobas 601 module (sera from Heidelberg, Tübingen and Ulm) or by recomWell SARS-CoV2 IgG ELISA (Mikrogen GmbH Martinsried, Germany) on a BEP III analyzer (sera from Freiburg). All measurements were performed according to the manufacturers´ instructions.

SARS-CoV-2 specific antibodies were further determined using indirect immunofluorescence on sub-confluent VeroE6 African green monkey cells in 96-well plates infected or not with SARS-CoV-2 (BavPat1/2020 strain, European Virus Archive). Cells were fixed with paraformaldehyde and either permeabilised (sera from Heidelberg, Tübingen and Ulm) or not (Freiburg). Fluorescence labelled secondary antibody (Goat anti-human IgG-AlexaFluor 488, Invitogen, Thermofisher Scientific or anti-human IgG-Cy3, Jackson ImmunoResearch) was used for detection. Plates with sera from Freiburg were subjected to fluorescence microscopy using a Zeiss Observer.Z1 inverted epifluorescence microscope (Carl Zeiss) equipped with an

© 2021 American Medical Association. All rights reserved.

AxioCamMR3 camera. Seropositivity was determined by visually comparing SARS-CoV-2-infected and uninfected cells incubated in parallel with the same person´s serum. Plates with sera from Heidelberg, Tübingen and Ulm were analyzed using a newly established semi-quantitative, semi-automated procedure described in detail in a pre-publication manuscript. Nine fluorescence images per well were acquired with a motorized Nikon Ti2 wide field microscope, automatically segmented, feature-extracted, analyzed and classified as described in Pape *et al.*[1]

Determination of neutralizing antibody titers (NT)

VeroE6 cells were cultured in Dulbecco's modified Eagle medium (DMEM, Life Technologies) containing 10% fetal bovine serum, 100 U/mL penicillin, 100 µg/mL streptomycin and 1% non-essential amino acids (complete medium). SARS-CoV-2 virus stocks were produced by amplification of the BavPat1/2020 strain (European Virus Archive) in VeroE6 cells. To generate the seed virus (passage 3), VeroE6 cells were infected with the original virus isolate, received as passage 2, at an MOI of 0.01. At 48 h post infection (h.p.i.), the supernatant was harvested and cell debris was removed by centrifugation at 800xg for 10 min. for production of virus stocks (passage 4). Virus titers were determined by plaque assay on VeroE6 cells, and virus stocks were stored in aliquots at -80°C.

Neutralizing antibodies were determined by infection of subconfluent VeroE6 African green monkey kidney cells with SARS-CoV-2 incubated or not with different concentrations of participants´ sera. Serial two-fold dilutions of sera were prepared in OptiMEM medium or PBS and incubated with SARS-CoV-2 for 1 h at 37°C or room temperature prior to infection of VeroE6 cells. For the analysis of sera from Heidelberg, Tübingen and Ulm, infection was scored at 20 h post infection (p.i.) by immunostaining of fixed cells using the anti-ds-RNA mouse monoclonal J2 antibody (Scicons, 1:1000) and a secondary anti-mouse HRP-coupled antibody (Merck, Darmstadt, Germany, 1:1000). The signal was developed using KPL SureBlueTM 3,3',5,5'-tetramethylbenzidine peroxidase substrate (Seracare, Milford, MA, USA) for 5 min and stopped by the addition of 0.5 M sulfuric acid. Absorbance was measured on a Tecan Sunrise plate reader (Tecan, Männedorf, Switzerland) at 450 nm with reference wavelength 620 nm. Data were normalized to the no-serum control (100%) and a mock-infected control (0%). In order to ensure reproducibility of results, one serum sample from a healthy donor and one sample from a symptomatic COVID-19 patient, collected at day 39 after onset of symptoms, were used in all experiments. Inhibitory dilution 50 (ID50), defined as serum dilution resulting in 50% reduction of normalized signal, was determined using the nonlinear regression function of GraphPad Prism software or a custom Excel template using the same four parameter dose response equation. For sera from Freiburg, virus incubated with sera or not was removed from VeroE6 cells after 1.5 h incubation at room temperature, and the cells were overlaid with 0.6% Oxoid-agar in DMEM, 20 mM HEPES (pH 7.4), 0.1% NaHCO3, 1% FBS and 0.01% DEAE-Dextran. Cells were formaldehyde fixed 48 h p.i. and stained with 1% crystal violet upon removal of the agar overlay. Plaque forming units (PFU) were counted manually. The number of plaques counted for serum-treated wells was compared to the average number of plaques in the untreated negative controls that were set to 100%.

Workflow of the serological analyses

The workflow of the serological analyses is shown eTable 1. Samples that tested positive in both assays were also subjected to neutralizing antibody titer (NT) determination. Samples that displayed only partial reactivity were further subjected to one of the following assays (i) ECLIA for antibodies against the viral N protein, (ii) ELISA for antibodies against the viral N protein, (iii) NT determination, or (iv) an in house Luminex-based assay detecting antibodies against N and S1 proteins17, depending on available sample volume and test site. All samples classified seropositive based on a positive result in at least two assays were additionally subjected to NT determination, if this had not been done during classification.

eResults

Descriptive characteristics of study population

The region of residence of study participants analyzed according to the three-digit postal code is depicted in eFigure 2. The majority of participants were residents of the study center towns Freiburg, Heidelberg, Tübingen, and Ulm or the adjacent regions, but the catchment area also included other regions of the federal state of Baden-Württemberg. Of 2482 adult study participants 553 (22.3%) were health care workers.

The number of reported siblings in the families was 0 in 460 (18.5%) families, 1 in 1423 (57.7%), 2 in 473 (19.2%), 3 in 96 (3.9%), 4 in 14 (0.6%) and 5 in 2 (0.1%) families (missing information in 14 families (0.1%)). 580 (23.4%) of the 2482 children analyzed attended exceptional child day care facilities during the study period. 237 of 2482 parents (9.5%) and 102 of 2482 children (4.1%) reported previous contacts with COVID-19 patients.

© 2021 American Medical Association. All rights reserved.

References

1. Pape C, Remme R, Wolny A, et al. Microscopy-based assay for semi-quantitative detection of SARS-CoV-2 specific antibodies in human sera. bioRxiv preprint doi: https://doi.org/10.1101/2020.06.15.152587.

© 2021 American Medical Association. All rights reserved.

eTable 1. Workflow of the serological analyses			
**ELISA Euroimmun IgG anti-S1**	**IF on SARS-CoV2 infected VeroE6 cells**	**Third Assay**	**Classification**
+	+	+	seropositive
+	-	+	seropositive
+	-	-	seronegative
?	+	+	seropositive
?	+	-	seronegative
?	-	+	seropositive
?	-	-	seronegative
-	+	+	seropositive
-	+	-	seronegative
-	-	n.d.	seronegative
? denotes borderline (OD ratio between 0.8 and 1.1); N.d., not done.			

© 2021 American Medical Association. All rights reserved.

eTable 2. Summary of mixed effect logistic regression models with the dependent variable "seropositivity"									
	**Model 1**			**Model 2**			**Model 3**		
	**Number of observations = 4964; number of families = 2482**			**Number of observations = 4964; number of families = 2482**			**Number of observations = 4722; number of families = 2361**		
Coefficient	log odds (standard error)	95% CI	***P*-Value**	log odds (standard error)	95% CI	***P*-Value**	log odds (standard error)	95% CI	***P*-Value**
Reference (intercept)	-17.81 (1.65)	-20.54–-14.91	<0.001	-17.26 (1.65)	-20.56–-14.15	<0.001	-16.89 (1.92)	-20.96–13.40	<0.001
Children aged 6-10 years				-0.59 (1.49)	-3.57–2.28	0.691	-0.61 (1.55)	-3.37–2.89	0.693
Parents	5.85 (0.95)	3.99–7.67	<0.001	5.51 (1.28)	3.01–7.87	<0.001	5.15 (1.35)	2.59–7.68	<0.001
Attending exceptional child day care - yes							-0.67 (1.52)	-5.25–1.87	0.660
Number of siblings							0.16 (0 .69)	-1.39–1.37	0.812
Sex - female							-0.17 (0.88)	-1.84–1.68	0.84
Previous contact to COVID-19 patient - yes							1.47 (1.16)	-1.36–3.64	0.207
CI, Confidence interval									
In model 1, the respective reference (intercept) is "children aged 1-10 years", in model 2 "children aged 1-5 years". For model 3, the coefficients "children aged 1-5 years", "attending exceptional child day care – no", "sex – male" and "contact to COVID-19 patient – no" are the respective reference (intercept) 3. All models incorporated the random effect "family" (see description in text).<br>The term "exceptional child day care" refers to emergency child day care during government-imposed lockdown because of the COVID-19 pandemic.									

© 2021 American Medical Association. All rights reserved.

lockdown because of the COVID-19 pandemic.

eTable 3. SARS-CoV-2 seroprevalence stratified according to the characteristic "exceptional child day care"

	**No./%**			
	**Children**	**Subgroup aged**	**Subgroup aged**	**Parents**
**Seropositivity**	**1- 10 years**	**1 - 5 years**	**6 - 10 years**	
Attending exceptional child day care – yes				
Negative	580	296	284	576
Positive	3	2	1	7
Percentage seropositivity	0.5%	0.7%	0.4%	1.2%
Attending exceptional child day care – no				
Negative	1863	814	1049	1841
Positive	19	7	12	41
Percentage seropositivity	1.0%	0.9%	1.1%	2.2%

For 34 (1.4%) households the information about exceptional child day care was not available.
The term "exceptional child day care" refers to emergency child day care during government-imposed lockdown because of the COVID-19 pandemic.

© 2021 American Medical Association. All rights reserved.

eTable 4. Covariates stratified according to seropositivity			
	**seropositivity**		
	**yes**	**no**	**Percent**
Children aged 1-5 years	9	1120	0.8
Children aged 6-10 years	13	1340	0.97
Parents	48	2434	1.97
Attending exceptional child day care			
Yes	3	580	0.52
No	19	1863	1.02
Unknown	0	17	0
Sex			
female	45	2987	1.51
male	25	1855	1.35
Unkown	0	52	0
Previous contact to COVID-19 patient			
Yes	21	318	6.60
No	49	4530	1.08
Unknown	0	46	0
Number of siblings			
0	6	914	0.66
1	43	2803	1.53
2	15	931	1.61
3	1	191	0.52
4	2	26	7.69
5	0	4	0
Unknown	3	25	12.00

The term “exceptional child day care” refers to emergency child day care during government-imposed

© 2021 American Medical Association. All rights reserved.

eFigure 1: Disposition of study participants

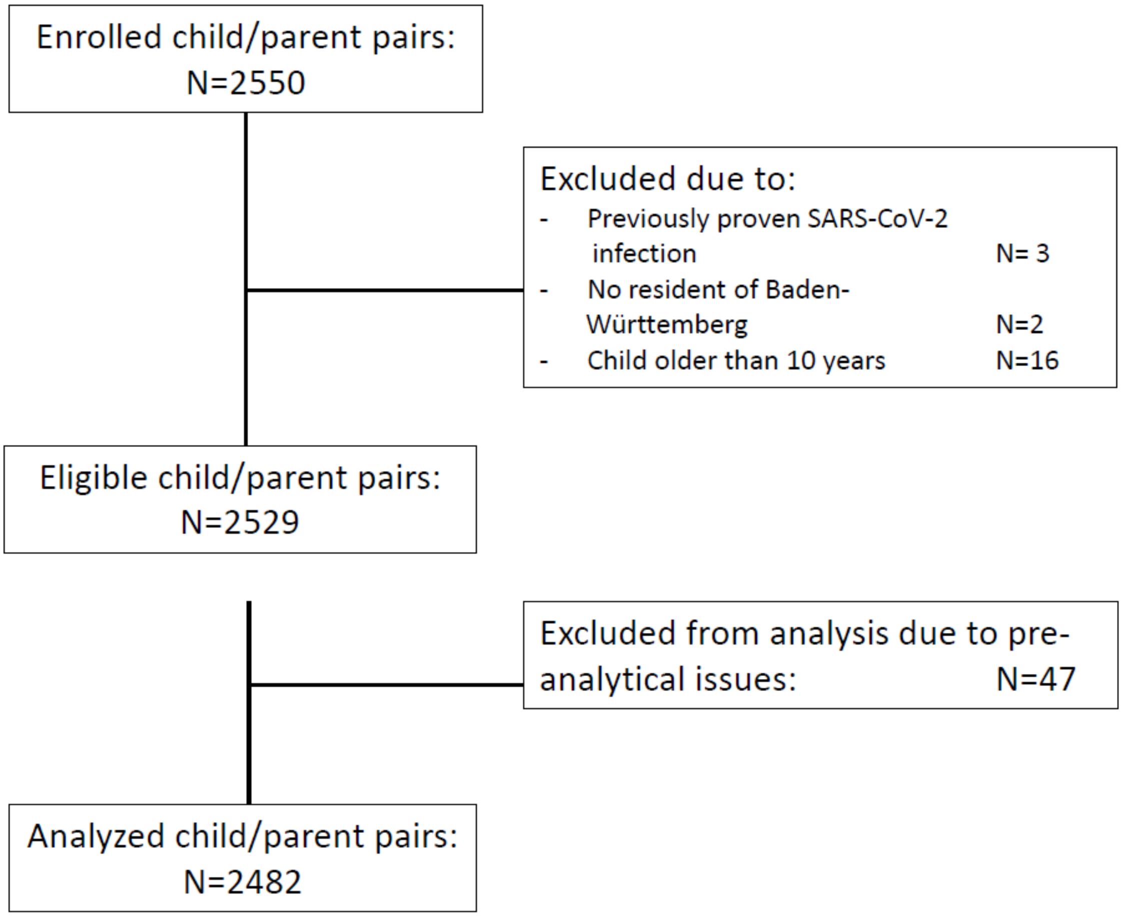

© 2021 American Medical Association. All rights reserved.

eFigure 2: Region of residence of study participants analyzed according to the three-digit postal code

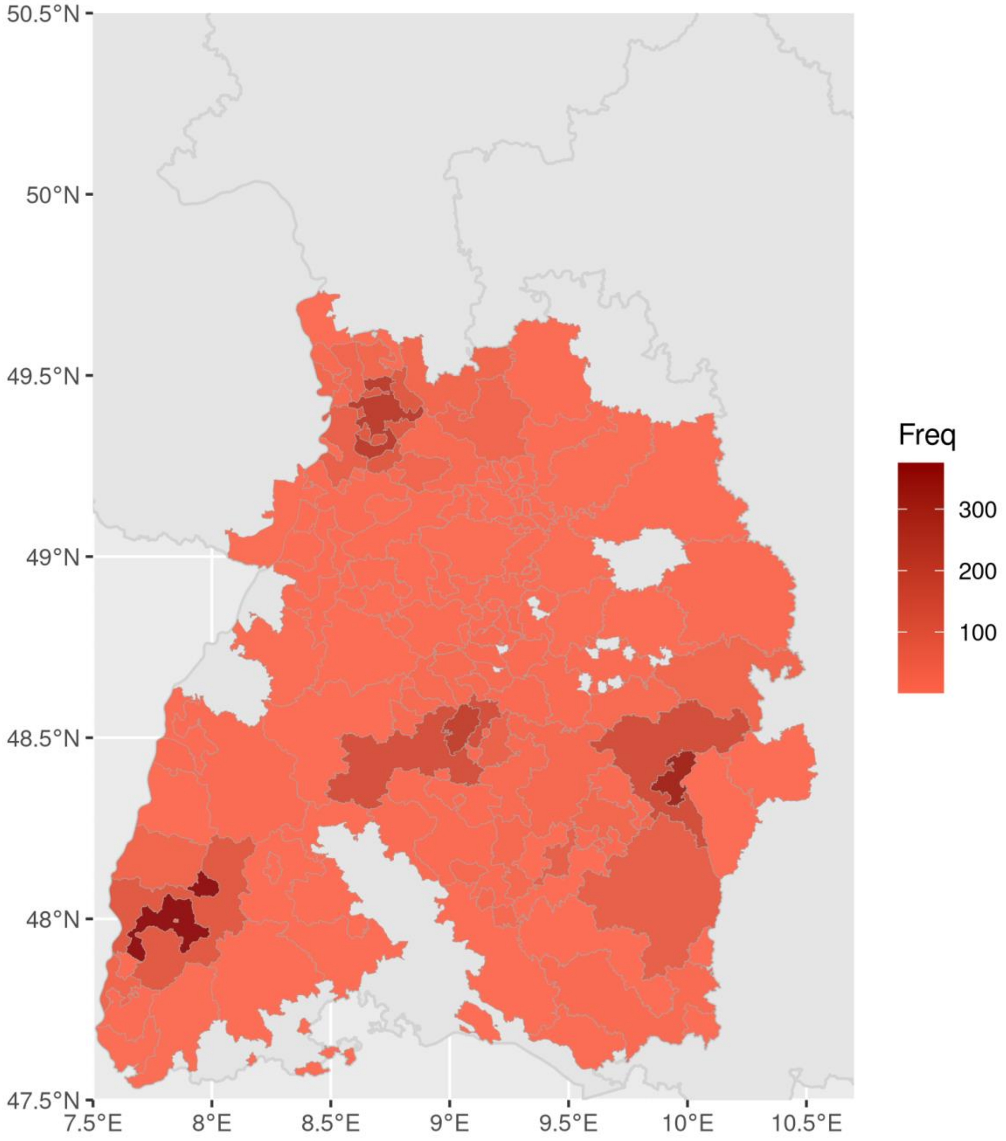

© 2021 American Medical Association. All rights reserved.
